# A combination of β-hydroxybutyrate and citrate ameliorates disease progression in a rat model of polycystic kidney disease

**DOI:** 10.1152/ajprenal.00205.2023

**Published:** 2023-12-14

**Authors:** Jacob A. Torres, Nickolas Holznecht, David A. Asplund, Tselmeg Amarlkhagva, Bradley C. Kroes, Juliette Rebello, Shagun Agrawal, Thomas Weimbs

**Affiliations:** Department of Molecular, Cellular, and Developmental Biology, University of California-Santa Barbara, Santa Barbara, California, United States

**Keywords:** β-hydroxybutyrate, ketosis, kidney, nephrolithiasis, polycystic

## Abstract

Our research has shown that interventions producing a state of ketosis are highly effective in rat, mouse, and cat models of polycystic kidney disease (PKD), preventing and partially reversing cyst growth and disease progression. The ketone β-hydroxybutyrate (BHB) appears to underlie this effect. In addition, we have demonstrated that naturally formed microcrystals within kidney tubules trigger a renoprotective response that facilitates tubular obstruction clearance in healthy animals but, alternatively, leads to cyst formation in PKD. The administration of citrate prevents microcrystal formation and slows PKD progression. Juvenile Cy/+ rats, a nonorthologous PKD model, were supplemented from 3 to 8 wk of age with water containing titrated BHB, citrate, or in combination to find minimal effective and optimal dosages, respectively. Adult rats were given a reduced BHB/citrate combination or equimolar control K/NaCl salts from 8 to 12 wk of age. In addition, adult rats were placed in metabolic cages following BHB, citrate, and BHB/citrate administration to determine the impact on mineral, creatinine, and citrate excretion. BHB or citrate alone effectively ameliorates disease progression in juvenile rats, decreasing markers of cystic disease and, in combination, producing a synergistic effect. BHB/citrate leads to partial disease regression in adult rats with established cystic disease, inhibiting cyst formation and kidney injury. BHB/citrate confers benefits via multiple mechanisms, increases creatinine and citrate excretion, and normalizes mineral excretion. BHB and citrate are widely available and generally recognized as safe compounds and, in combination, exhibit high promise for supporting kidney health in polycystic kidney disease.

**NEW & NOTEWORTHY** Combining β-hydroxybutyrate (BHB) and citrate effectively slows and prevents cyst formation and expansion in young Cy/+ rats using less BHB and citrate than when used alone, demonstrating synergy. In adult rats, the combination causes a partial reversal of existing disease, reducing cyst number and cystic area, preserving glomerular health, and decreasing markers of kidney injury. Our results suggest a safe and feasible strategy for supporting kidney health in polycystic kidney disease (PKD) using a combination of BHB and citrate.

## Graphical abstract

**Figure:**
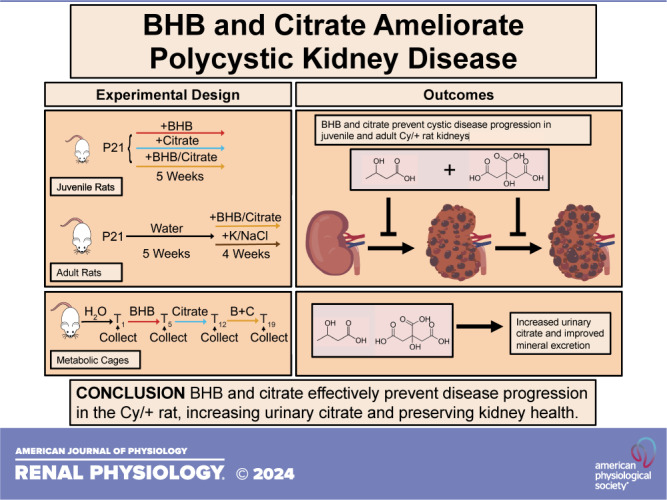

## INTRODUCTION

Autosomal dominant polycystic kidney disease (ADPKD), caused by mutations in the polycystin-1 (*PKD1*) or polycystin-2 (*PKD2*) genes, is the most common life-threatening monogenic disease, affecting at least 1:1,000 individuals worldwide (1). Currently, the only approved medication to slow ADPKD progression, tolvaptan, is not readily available or desirable for many individuals due to cost (2), eligibility restrictions, side effects, and toxicities (3). Identifying more effective, accessible, affordable, and safe alternative treatment options is therefore of great interest.

ADPKD causes the formation of large fluid-filled cysts that progressively replace healthy kidney tissue, causing kidney function decline. Complications of ADPKD include an increased risk of developing kidney stones (4, 5). Kidney stones grow from tubular microcrystals that precipitate within supersaturated fluids. Genetic ablation of *Pkd1* or *Pkd2* in adult rodents does not, in itself, result in rapid polycystic kidney disease (PKD) but can be accelerated following kidney insults (6, 7). Our laboratory has previously shown that tubular calcium oxalate and calcium phosphate microcrystals can trigger cystogenesis and accelerate disease progression in a PKD rat model (*Anks6* mutant, Cy/+ rats) (5). Citrate in the tubule lumen is an essential natural defense against the formation of calcium-derived microcrystals, acting as a calcium chelator. Increasing citrate intake from food sources, supplements, or prescription drugs is a common strategy to facilitate the suppression of microcrystal formation in recurrent kidney stone formers (8–10). Tanner et al. (11–13) previously demonstrated that administration of potassium citrate to the Cy/+ rat effectively ameliorated PKD progression, although not recognizing that the mechanism may involve the removal of tubular microcrystals. We have demonstrated that oral citrate administered in drinking water effectively eliminates microcrystals in PKD kidneys (5).

The Cy/+ rat is a nonorthologous model of ADPKD where male rats rapidly develop proximal tubule-derived cysts until 8 wk of age and then exhibit slow kidney function decline until 6 mo. Female rats develop a less severe phenotype, survive past 1 yr of age, show slower renal function decline, and are resistant to the formation of microcrystals (5, 14). We chose to use male Cy/+ rats for this study primarily for their ability to form kidney microcrystals that citrate supplementation can remove.

In addition, mutations in *Pkd1/Pkd2* alter cellular metabolism, shifting cells toward a glycolytic phenotype with impaired fatty acid oxidation (15, 16) accompanied by alterations in mitochondrial structure and function, increased oxidative stress, and altered gene expression (15, 17–19). Addressing this metabolic switch, we have previously reported that dietary interventions that induce a state of ketosis, including time-restricted feeding (TRF), fasting, and a ketogenic diet, strongly inhibit cyst growth in rodent and feline models of PKD (20). We have further reported that supplementation in drinking water with the significant ketone produced by the liver during ketosis, β-hydroxybutyrate (BHB), mimicked the beneficial effects of dietary ketosis (20). A recent randomized, controlled clinical trial based on these preclinical results also suggested that a ketogenic diet improves renal function in individuals with ADPKD (21).

In this study, we test if a combination of BHB and citrate may provide enhanced benefit in the Cy/+ rat to a greater extent than when either BHB or citrate are supplemented alone. We hypothesized that BHB and citrate may help prevent disease progression by acting on distinct mechanisms of disease progression: mimicking the effects of ketosis and preventing injury from tubule microcrystals, respectively. To test this hypothesis, we supplemented titrated amounts of BHB and citrate to determine their minimal effective dose and then combined them. BHB and citrate possess a strong safety profile, are natural products of metabolism, and are inexpensive. If effective, BHB and citrate would provide an accessible and safe option for those affected by ADPKD. Our results indicate that BHB and citrate, at reduced doses, may act synergistically to prevent disease progression in juvenile rats and ameliorate established kidney disease in adult rats, reducing cystic area, preventing cystogenesis, kidney injury, increasing citrate excretion, and maintaining glomerular health.

## MATERIALS AND METHODS

### Animal Experiments

The Hannover Sprague-Dawley (Cy/+) rat was used for all experiments (14). All rats were housed in the animal resource center at the University of California-Santa Barbara using a 12:12-h light-dark cycle with ad libitum access to food, water, and enrichment. All animal experiments were performed with the approval of the University of California-Santa Barbara Institutional Animal Care and Use Committee. Rats were weaned at *postnatal day 21*, separated by sex, group housed, and randomly assorted. Experimental replicates from different litters were used for each treatment and analysis. Sample sizes were determined based on previous experimental observations using this model. Food and water intakes were measured throughout all experiments, and rat weights were measured weekly. BHB was measured using a blood meter (Precision Xtra, Abbott) and glucose with a glucometer (Contour Next EZ, Bayer) before rats were anesthetized using a combination of 200 mg/kg:20 mg/kg ketamine-xylazine followed by cervical dislocation before tissue removal. Tissue samples were snap-frozen in liquid nitrogen following removal for later analysis. Serum samples were collected by cardiac puncture, transferred to a Microtainer tube (Cat. No. B-D365967, BD), separated by centrifugation, and snap-frozen in liquid nitrogen. During the experimental period, the lead researcher was responsible for sorting animals into experimental cages. Both PKD and wild-type rats were housed together, resulting in a semi-blinded experiment in which researchers involved in feeding and watering animals were blinded to the specific genotypes of animals within treatment groups during the experimental period. A detailed description of the animals used for each experiment is provided in the Animal Use Table in the Supplemental Material.

#### β-Hydroxybutyrate.

An unflavored sodium/potassium salt of d/l-BHB (KetoForce, Ketosports) was added to water ad libitum for all BHB experiments with a concentration of 4.2% and then halved to 2.1% and 1% for titration experiments. These concentrations were rounded off and labeled as 160, 80, and 40 mM, respectively. We have previously tested a comparable salt control to our 160 mM dose in the study by Torres et al. (20). We showed that salt alone did not have an effect and, therefore, could not justify a repeat of that experiment in this study.

#### Citrate.

A combination of tripotassium citrate (CAS No. 6100-05-6, Sigma-Aldrich) and citric acid (CAS No. 77-92-9, Sigma-Aldrich) was used to make citrate solutions as previously described (11). The concentrations of 55 mmol/L tripotassium citrate/67 mmol/L citric acid, 27.5 mmol/L tripotassium citrate/33.5 mmol/L citric acid, and 13.75 mmol/L tripotassium citrate/16.75 mmol/L citric acid were rounded off and labeled as 120, 60, and 30 mM, respectively.

#### BHB and citrate.

BHB and citrate were made by combining the mixtures described earlier in their respective amounts indicated by their millimolar concentration. Adult rats received a 1% BHB and 27.5 mM tripotassium citrate/33.5 mM citric acid solution (40/60) and a solution containing 2.1% BHB and 13.75 mM tripotassium citrate/16.75 mM citric acid (80/30). As a control, rats were supplemented with a salt solution of potassium/sodium chloride lacking BHB and citrate of 22 mM sodium/98 mM potassium (40/60 salt) or 46.5 mM sodium/73.5 mM potassium (80/30 salt), respectively.

#### Metabolic cage experiments.

To analyze urine composition, adult rats aged 10–12 wk were housed in metabolic cages (Cat. No. 526715, Tecniplast) using a chiller (Cat. No. 72906, Tecniplast) to preserve urine samples during collection. Rats were given ad libitum food and water access during the experiment. Collection occurred on *time 0* to establish a 24-h baseline, followed by 4.2% BHB administration in water for 3 days and a 24-h collection period. Rats were then returned to their home cage and given plain water for a 3-day “washout” period before they received 120 mM citrate in water for 3 days and a subsequent 24-h urine collection, followed by an additional 3-day “washout” period, administration of 2.1% BHB/13.75 mmol/L tripotassium citrate/16.75 mmol/L citric acid solution for 3 days, and a 24-h urine collection.

#### Glomerular health scoring.

Kidney sections were stained for podocin, and all glomeruli were scored using the following rubric: 0 = no obvious morphological changes (normal); 1 = morphological change, e.g., changes in shape and structure; and 2 = morphological changes as well as decreased filling of the glomeruli space and an increase in distance between Bowman’s capsule and podocin.

#### Creatinine.

Serum creatinine was calculated using a QuantiChrom creatine assay kit (Cat. No. DICT-500, BioAssay Systems).

### mtDNA Quantitative PCR

Mitochondrial (mt)DNA quantification was done as previously described by Zhang et al. (22). DNA was extracted from 25 mg of frozen tissue using a Zymo-spin TM IICR column mini-prep (Cat. No. D4068, Zymo Research), purified using sodium acetate, ethanol precipitated, washed with 70% ethanol, air-dried, resuspended in sterile TE buffer, diluted to 3 ng/µL, and then subjected to quantitative PCR (Cat. No. 6020, Promega).

The following primers were used: 195-bp product rat clusterin (TRPM-2), forward 5′- GGTGTACTTGAGCAGAGCGCTATAAAT-3′ and reverse 5′- CACTTACCCACGGCAGCTCTCTAC-3′ and 235-bp product rat mitochondria cytochrome *b*, forward 5′- CCTCCCATTCATTATCGCCGCCCTTGC-3′ and reverse 5′- GTCTGGGTCTCCTAGTAGGTCTGGGAA-3′.

PCR conditions were as follows: 1 cycle of 94°C for 5 min, 35 cycles of 94°C for 15 s, 63°C for 45 s, and 72°C for 60 s, 1 cycle of 94°C for 60 s, 1 cycle of 72°C for 30 s, and 1 cycle of 95°C for 30 s.

The mitochondrial number was determined by obtaining threshold cycle (C_T_) values for mtDNA and nuclear DNA and then calculating ΔC_T_ = (nuclear DNA C_T_ – mtDNA C_T_). The relative mitochondrial number was calculated using (mitochondrial number = 2 × 2^ΔCT^).

### Statistics

When appropriate, unpaired and paired two-tailed *t* tests were used to compare differences between groups. One-way ANOVA followed by an ad hoc Tukey’s test was used for multiple comparisons. Statistical analysis was performed using Prism 8 (GraphPad). Individuals were used as the experimental unit. Cage cohorts were quantified for food and water intake analysis. For analyses that used fewer than an entire group of experimental animals, animals were chosen randomly for inclusion. Multiple litters were used for each experimental condition.

### Antibodies

The following primary antibodies were used: rabbit anti-smooth muscle actin (SMA; Cat. No. ab5694, Abcam, RRID:AB_2223021) diluted 1:200 for immunofluorescence; mouse anti-Ki67, clone B56 (RUO) (Cat. No. 550609, BD Biosciences, RRID:AB_393778), diluted 1:200 for immunofluorescence; rabbit anti-peroxisome proliferator-activated receptor-γ coactivator-1α (PGC-1α; Cat. No. NBP1-04676, Novus, RRID:AB_1522118) diluted 1:1,000 for Western blot analysis; rabbit anti-podocin (Cat. No. PA5-79757, Thermo Fisher Scientific, RRID:AB_2746872) diluted 1:200 for immunofluorescence; rabbit anti-p44/p44 MAPK-Erk1/2 (137F5, Cat. No. 4695, Cell Signaling Technology, RRID:AB_390779) diluted 1:1,000 for Western blot analysis; rabbit anti-p44/p44 MAPK phospho-(Thr^202^/Tyr^204^)-Erk1/2 (D13.14.4E, Cat. No. 4370, Cell Signaling Technology, RRID:AB_2315112) diluted 1:1,000 for Western blot analysis; rabbit anti-phosphorylated (p)STAT3 (Tyr^705^) (D3A7, Cat. No. 9145, Cell Signaling Technology, RRID:AB_2491009) diluted 1:1,000 for Western blot analysis; mouse anti-total STAT3 (124H6, Cat. No. 9139, Cell Signaling Technology, RRID:AB_331757) diluted 1:1,000 for Western blot analysis; mouse anti-actin (Cat. No. A5441, Sigma-Aldrich, RRID:AB_476744) diluted 1:10,000 for Western blot analysis; and goat anti-kidney injury molecule-1 (KIM-1; Cat. No. AF3689, R&D Systems, RRID:AB_2116557) diluted 1:1,000 for Western blot analysis.

The following secondary antibodies were used: goat anti-rabbit Alexa Fluor 594 (Cat. No. A-11012, Thermo Fisher Scientific, RRID:AB_2534079) diluted 1:1,000 for immunofluorescence and goat anti-mouse Alexa Fluor 594 (Cat. No. A-11005, Thermo Fisher Scientific, RRID:AB_2534073) diluted 1:1,000 for immunofluorescence.

### Western Blot Analysis

Approximately 10 mg of snap-frozen tissue samples were lysed in 200 µL of SDS lysis buffer (4% SDS, 100 mM Tris·HCl (pH 6.8), 20% glycerol, 1:1,000 protease inhibitor cocktail (pepstatin E110, leupeptin E18, antipain E13, Chemicon; benzmidine, Sigma-Aldrich; Traysol, Bayer), and 1:100 phosphatase inhibitor cocktails 2 and 3 (Cat. Nos. P5726 and P0044, Sigma-Aldrich) followed by heating at 100°C. Protein lysates (25 µg) were subjected to SDS-PAGE using 10% acrylamide gels, transferred onto nitrocellulose membrane, incubated overnight with primary antibodies, washed, incubated with secondary antibody, washed, and then imaged using an Azure 600 (Azure Biosystems). Western blot images were quantified via densitometry using FIJI (ImageJ) (23).

### Microscopy

All control and experimental samples were imaged in the same session for use in figures and quantification. Bright-field images were white balanced and altered to normalize color using Photoshop (Adobe). The contrast and brightness of control and experimental images were adjusted similarly within the figures.

### Immunofluorescence

Deparaffinized slides were subjected to pressure cooker antigen retrieval with 10 mM sodium citrate (pH 6.0) and then blocked [1% BSA, 0.1% Triton X-100, and 0.1% fish skin gelatin in Tris-buffered saline (TBS) with Tween 20 (TBST)] in a humid chamber at 37°C for 60 min. Primary antibodies were mixed with blocking buffer and incubated on sections overnight at 4°C. Slides were then washed in TBST and incubated in 0.1% Sudan black B in 70% ethanol for 20 min. Slides were then incubated in a secondary antibody in a humid chamber protected at 37°C. Slides were then washed, fixed with 10% NBF, washed in TBST, stained with DAPI in TBS, rinsed in TBST, and mounted using Prolong Gold (Cat. No. P36930; Thermo Fisher). No primary and secondary only controls were used as controls to control for the specificity of antibodies.

### Histology

Kidney tissue samples were excised from rats, immediately rinsed in PBS, weighed, and then fixed in 10% neutral buffered formalin for 24 h at ambient temperature, followed by paraffinization. Sections (5 µm) on Superfrost Plus slides (Cat. No. 12-550-15, Fisher Scientific) were used for all histology and immunofluorescence applications.

#### Hematoxylin and eosin.

Rehydrated samples were placed in hematoxylin solution for 1 min, rinsed in running tap water, placed into eosin for 45 s, dipped 10 times in 2 × 95% and 2 × 100% ethanol and then 2 × 5 min in xylenes, and mounted using Permount (Cat. No. SP15-100, Fisher Scientific).

#### Collagen.

Deparaffinized kidney samples were stained using a Sirius Red/Fast Green Collagen Staining kit (Cat. No. 9046, Chondrex).

### Quantification

Quantification occurred in a semi-blinded manner in which researchers quantified animals without knowledge of specific treatments for each animal being counted. Researchers only had access to the animal ID without knowledge of the treatment when quantifying but were, however, aware of the overall experimental design and potential outcomes.

#### Fibrosis.

Ten images from cortical regions of sirius red-stained sections were imaged at ×100 magnification for quantification. Using Photoshop (Adobe), a grid was placed over each image, and intersections with sirius red stain were counted as positive, with other intersections counted as negative with intersections overlaid on negative space excluded from the total number of potential intersections. The total number of positive intersections was divided by the total possible number of intersections to obtain the percentage of fibrosis.

#### Cystic index.

Ten cortical images of hematoxylin and eosin-stained sections were imaged at ×100 magnification for quantification. Using Photoshop (Adobe), a grid was placed over each image, and intersections overlaid on cysts were counted as positive, with other intersections as negative. Empty space intersecting outside of the tissue was excluded from the total number of potential intersections. The total positive intersections were divided by the total possible number of intersections to obtain the cystic index.

#### Smooth muscle actin.

Ten images from cortical regions were taken from each kidney of SMA-stained sections at ×200 magnification. Using Photoshop (Adobe), a grid was placed over each image, and intersections with SMA stain were counted as positive and other intersections counted as negative, with intersections overlaid on negative space excluded from the total number of potential intersections. The total number of positive intersections was divided by the total possible number of intersections to obtain the percentage of the SMA-positive area.

#### Ki67.

Five images of Ki67-stained sections were imaged at ×200 from cortical regions. At least 1,000 cells were counted for each animal. Cell number was determined with DAPI using FIJI (ImageJ) (23) to automate cell counting. Ki67-positive cells were manually counted and classified by location as interstitial (existing outside of tubules and cysts) or cystic/tubular (existing in cysts or tubules). The number of positive cells from all five images for each location was divided by the number of cells counted to obtain the percentage of Ki67-positive cells.

#### Cyst number and cyst size.

Whole kidney sections were imaged using a dissecting scope, and individual cysts were manually counted using the wand tool in FIJI (ImageJ).

#### Depictions of synergism.

Figures that investigate possible synergism between BHB and citrate when used in combination were derived using descriptions from Tallarida (24). To depict synergism, the effect size of PKD parameters was plotted relative to the amount of BHB and citrate administered. The greatest effect measured using the least amount of BHB and citrate was then plotted on an isobologram to depict if the combination of BHB/citrate produces an effect with a reduced dose.

### Ultra-High Pressure Liquid Chromatography Mass Spectrometry

l-BHB and d-BHB isomers were measured in serum using ultra high-pressure liquid chromatography mass spectrometry (UHPLC-MS) following derivatization to separate isomers.

#### Chemicals and materials.

The following chemicals and materials were used: d-BHB (CAS No. 625-72-9, Cat. No. 54920, Sigma-Aldrich), l-BHB (CAS No. 6168-83-8, Cat. No. 54925, Sigma-Aldrich), sodium *d*_4_-dl-BHB (Na *d*_4_-dl-BHB; CAS No. 1219804-68-8, Cat. No. 14158, Cayman Chemical), *S*-1-(2-pyrrolidinyl methyl)-pyrrolidine (*S*-PMP; CAS No. 51207-66-0, Cat. No. P12411G, Sigma-Aldrich), triphenylphosphine (TPP; CAS No. 603-35-0, Cat. No. 140420250, Sigma-Aldrich), and 2,2′-dipyridyl disulfide (DPDS; CAS No. 2127-03-9, Cat. No. D11145G, Sigma-Aldrich).

##### Solvents.

The following solvents were used: LCMS grade water with 0.1% formic acid (FA; vol/vol) (CAS Nos. 7732-18-5 and 64-18-6, Cat. No. LS118-4, Fisher Scientific), LCMS grade acetonitrile (ACN) with 0.1% FA (vol/vol) (CAS No. 75-05-8, 64-18-6, Cat. No. LS120-4, Fisher Scientific), LCMS grade ACN (CAS No. 75-05-8, Cat. No. A995-4, Fisher Scientific), LCMS grade methanol (CAS No. 67-56-1, Cat. No. A456-4, Fisher Scientific), and MilliQ water (CAS No. 7732-18-5, Millipore Sigma).

##### Materials.

The following materials were used: 96-well 0.2-μm polypropylene vacuum filtration plate (Cat. No. PI90036, Thermo Scientific).

##### Software.

MassLynx (v.4.1, Waters) software was used to acquire and process data.

##### Methods.

The following methods were modified from previously published methods (Tsutsui et al., 25). For UPLC-ESI-QToF, a Waters Acquity H-class UPLC system coupled to a Waters Xevo G2-XS QToF Quadrupole Time-of-Flight Mass Spectrometer was used for the analysis, and the column used was a Waters BEH C18 UPLC column (1.7 μm, 100 × 2.1 mm). The following parameters were used for the analysis:

- UPLC parameters. Injection volume, 1.00 µL; column temperature, 40°C; and flow rate, 0.350 mL/min.
- Gradient profile. 0.00 min, 100% H_2_O-FA, 0% ACN-FA; 5.00 min, 90% H_2_O-FA, 10% ACN-FA; 8.00 min, 0% H_2_O-FA, 100% ACN-FA; 9.00 min, 95% H_2_O-FA, 5% ACN-FA; and 11.01 min, 100% H_2_O-FA, 0% ACN-FA. The autosampler temperature was 10°C.
- QToF parameters. Polarity: positive, analyzer: sensitivity mode, capillary voltage: 0.50 kV, sampling cone voltage: 30 V, source temperature: 120°C, source offset: 80, desolvation temperature: 350°C, cone gas flow: 50 L/h, desolvation gas flow: 1,000 L/h, LM resolution: 10, HM resolution: 15, and sample infusion flow rate: 10 µL/min.

#### Standard solutions.

d-BHB and l-BHB were made at 1,000.00 mg/L in ACN. The internal standard Na *d*_4_-dl-BHB was made at 10.00 mg/L in a solution of 90:10 ACN:MilliQ water (vol/vol), and Na dl-AHB was made at 1,000.00 mg/L in the same 90:10 solution. The *S*-PMP, TPP, and DPDS reagents were each made to 20.00 mM in ACN.

#### Working solutions.

d-BHB and l-BHB were made through 10-fold dilutions of the standards with ACN to make solutions with 100.00, 10.00, 1.00, and 0.10 mg/L concentrations.

#### Calibration solutions.

The calibration range for d-BHB and l-BHB ranged from 1 to 1,000 μg/L. To a microcentrifuge tube was added an appropriate aliquot of the analyte, 100.0 µL of internal standard, 100.0 µL of *S*-PMP, 100.0 µL of TPP, and 100.0 µL of DPDS, and samples were then diluted with the appropriate amount of ACN to reach a final volume of 1.00 mL. The solution was then vortexed and allowed to react at room temperature overnight to ensure a complete reaction. Once reacted, 100.0 µL of calibration solution were transferred to an LCMS vial and diluted with 900 µL of a solution of 98:1.6:0.4 H_2_O-FA:MeOH:ACN (vol/vol/vol). The LCMS vial was then vortexed and loaded into the instrument for analysis. Calibration solutions were made in triplicate.

#### Sample preparation.

To a microcentrifuge tube was added 10.00 µL of the sample (serum or urine), 100.0 µL of internal standard, and 890 µL of ACN. The tube was then vortexed and centrifuged for 5 min at 15,000 rpm, and the supernatant was filtered through a 96-well 0.2-μm polypropylene vacuum filtration plate. The filtered sample was then transferred to a microcentrifuge tube, and the solvent was evaporated in a vacuum centrifuge at 40°C. To the remaining residue was added 700 µL of ACN, 100.0 µL of S-PMP, 100.0 µL of TPP, and 100.0 µL of DPDS. The solution was then vortexed and allowed to react at room temperature overnight to ensure a complete reaction. Once reacted, 100.0 µL of sample solution were transferred to an LCMS vial and diluted with 900 µL of 98:1.6:0.4 H_2_O-FA:MeOH:ACN (vol/vol/vol). The LCMS vial was then vortexed and loaded into the instrument for analysis.

#### Calibration curve.

Calibration curves were constructed for both d-BHB and l-BHB by plotting the average peak area ratios of d-BHB and l-BHB to the internal standard against the corresponding concentration of analyte and then using the method of least squares linear regression to compute the equations of the lines. The calibration was acceptable with an *R*^2^ value of ≥0.995.

##### Quantification of d-BHB and l-BHB from samples.

The concentrations of d-BHB and l-BHB from the samples were calculated by determining the peak area ratios of d-BHB and l-BHB to the internal standard and then using the equations of the lines to determine the corresponding concentration.

### Inductively Coupled Plasma-Optical Emission Spectrometry

#### Chemicals and materials.

ICP grade 70% nitric acid (CAS No. 7697-37-2, Cat. No. A509P212) and ICP grade 30% hydrogen peroxide (CAS No. 7722-84-1, Cat. No. 02003185) were purchased from Fisher Scientific. ICP grade 100 μg/mL multi-analyte (Ca, K, Mg, Na) custom solution in 5% nitric acid, ICP grade 100 μg/mL yttrium in 2% nitric acid (CAS. No. 7440-65-5, Cat. No. MSY-100PPM-125ML), and ICP grade 5% nitric (CAS No. 7697-37-2, Cat. No. IV-ACID-BLANK-1L) were purchased from Inorganic Ventures.

MilliQ water was used throughout the analysis (CAS No. 7732-18-5, Millipore Sigma).

#### ICP-OES instrument and parameters.

An Agilent 5800 Inductively Coupled Plasma-Optical Emission Spectrometer was used for the analysis with the following parameters: viewing mode: radial, RF power: 1.20 kW, viewing height: 8 mm, read time: 5 s, stabilization time: 15 s, replicates: 3, pump speed: 12 rpm, nebulizer flow: 0.70 L/min, plasma flow: 12.0 L/min, and aux flow: 1.00 L/min.

The following wavelengths (in nm) were used to monitor the analytes: sodium: 589.595, 588.995, and 818.3; magnesium: 279.553, 280.270, and 285.213; potassium: 766.490, 771.531, and 404.721; calcium: 393.366, 396.847, and 422.673; and yttrium: 377.433, 371.030, 362.073.

##### Software.

ICP Expert Pro software (v.7.5.4.11997) was used to acquire and process data.

#### Calibration solutions.

Calibration solutions were made from 100 to 0.1 μg/mL by serially diluting the multi-analyte standard with 5% nitric acid in 50-mL polypropylene tubes. A 100.0-µL aliquot of 100 μg/mL yttrium was added as an internal standard.

#### Calibration curve.

Calibration curves were constructed for each analyte by plotting the average wavelength intensity ratio of each analyte to yttrium against the corresponding concentration of the analyte. The least squares linear regression method was then used to compute the equations of the lines of best fit. The calibration curve for each analyte was acceptable, with an *R*^2^ value of ≥0.995.

#### Sample preparation.

A 200.0-µL aliquot of urine was added to a tared digestion vessel, and its mass was recorded. The vessel was tared again, a 100.0-µL aliquot of the internal standard was added, and its mass was recorded. An aliquot of 200.0 µL of 70% nitric acid was then added to the vessel, and the sample sat at room temperature uncapped for 1 h to predigest. The vessel was then placed on a heating block at 110°C, and the sample was allowed to digest for 2 h. After being heated, the vessel was removed from the block and cooled to room temperature. An aliquot of 50.0 µL of 30% hydrogen peroxide was added to the sample, and the vessel was returned to the heating block at 110°C. The sample was digested uncapped for 14 h, after which the vessel was removed and brought to room temperature and its mass was recorded. The sample was then diluted to 10 mL with 5% nitric acid, and its final mass was recorded and capped until analysis.

### Quantification of Analytes from Samples

The concentrations of the analytes from the urine samples were calculated by determining the wavelength intensity ratios of each analyte to yttrium and then using the equations of the lines to determine the corresponding concentration.

## RESULTS

### BHB Ameliorates PKD in a Dose-Dependent Fashion in Juvenile Rats

**Figure 1.**
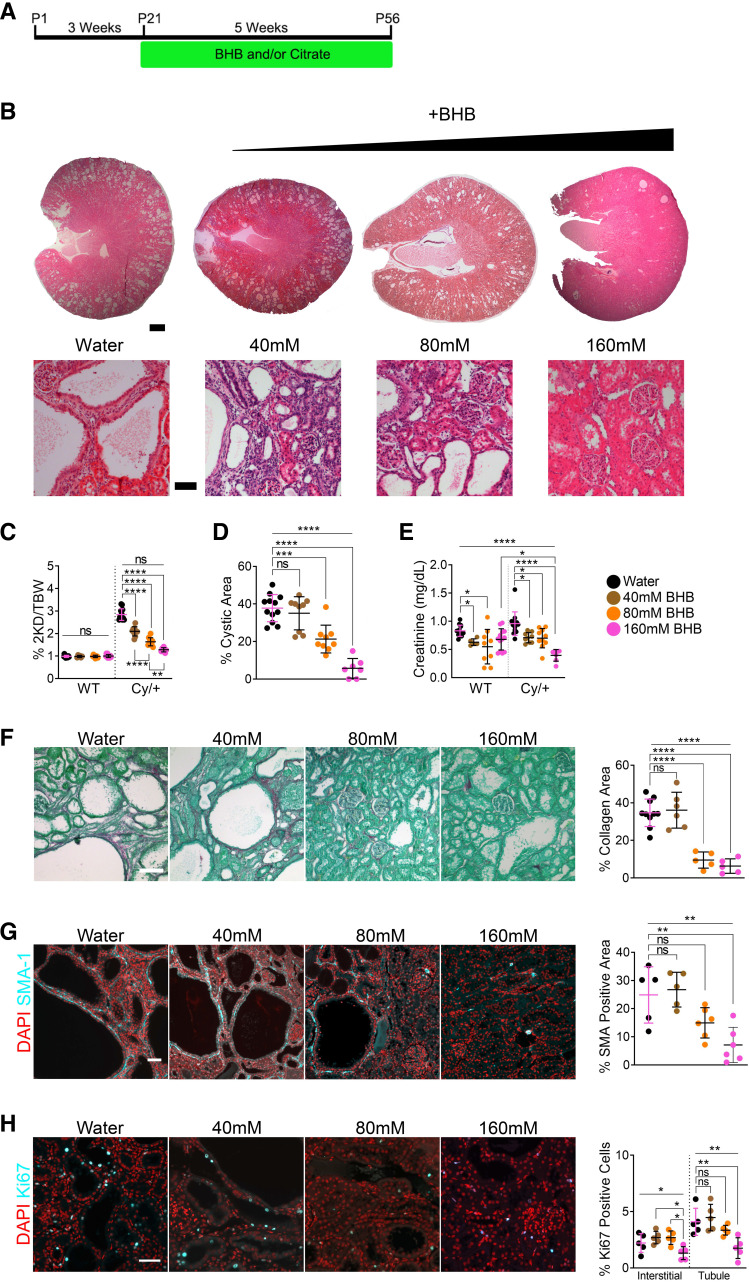
β-Hydroxybutyrate (BHB) ameliorates polycystic kidney disease (PKD) in a dose-dependent manner. *A*: treatment scheme for all juvenile BHB and/or citrate experiments (Figs. 1–3). *B*: hematoxylin and eosin-stained kidneys from 8-wk male wild-type and Cy/+ rats supplemented with water or increasing concentrations of BHB salts in drinking water. Scale bar = 1 mm and 100 µm, respectively. *C*: 2-kidney to the bodyweight of water and BHB-supplemented male wild-type and Cy/+ rats. *D*: cystic area of water and BHB-supplemented male Cy/+ rats. *E*: serum creatinine of male wild-type and Cy/+ rats. *F*: Sirius Red and Fast Green stain and quantification of water and BHB-supplemented male Cy/+ rats. Scale bar = 50 µm. *G*: smooth muscle actin (SMA-1) immunofluorescence and quantification of water and BHB-supplemented male Cy/+ rats. Scale bar = 50 µm. *H*: Ki67 immunofluorescence stain and quantification of water or BHB-supplemented male Cy/+ rats. Scale bar = 50 µm. Wild type: *n* = 12 (Water), *n* = 6 (40 mM BHB), *n* = 9 (80 mM), *n* = 12 (160 mM BHB). Cy/+: *n* = 12 (Water), *n* = 10 (40 mM BHB), *n* = 10 (80 mM), *n* =7 (160 mM BHB). (Standard deviation and means represented. One-way ANOVA followed by ad hoc Tukey’s test was used for multiple comparisons. *P < 0.05, **P < 0.01, ***P < 0.001, ****P < 0.0001).

We have previously reported that 160 mM d/l-BHB supplemented in drinking water for 5 wk almost completely halts PKD progression (20) and that administration of citrate in water can effectively remove microcrystals that exacerbate PKD in Cy/+ rats (5). Here, our study aimed to titrate the effective dose of BHB and citrate and test whether their combination can again alter PKD progression in Cy/+ rats (Fig. 1*A*). For our experimental analyses, we focused primarily on male Cy/+ rats. Male Cy/+ rats exhibit more aggressive kidney disease, have the propensity to form microcrystals, and succumb to advanced kidney disease at ∼6 mo of age. In contrast, female rats survive 12 mo or more (14) and are inherently resistant to microcrystal formation (5), precluding us from studying the effects of microcrystal-induced kidney injury. We included female rats in our study (Supplemental Material) for completeness, allowing for appreciation of the mechanism of BHB and citrate outside of their effect on microcrystal inhibition.

Like our previous experiments (20), we administered BHB to juvenile Cy/+ rats from *postnatal day 21* to *postnatal day 56* using a 160 mM starting concentration and halved it twice to 80 mM and 40 mM, respectively. BHB significantly reduced cystic disease in a dose-dependent manner (Fig. 1*B*), including a reduction in two kidney-to-body weight ratios (Fig. 1*C* and Supplemental Fig. S2*A*), cystic area (Fig. 1*D* and Supplemental Fig. S2*B*), cyst number (Supplemental Fig. S1*C*), and cyst size (Supplemental Fig. S1*D*), suggesting that BHB affects both cystogenesis and cyst expansion. BHB also reduced serum creatinine, suggesting improved creatinine clearance (Fig. 1*E*).

We examined disease hallmarks of PKD and found that 80 and 160 mM BHB strongly inhibited collagen deposition (Fig. 1*F* and Supplemental Fig. S2*C*) and myofibroblasts (Fig. 1*G* and Supplemental Fig. S2*D*). Ki67 staining revealed decreased proliferation with 160 mM BHB in tubule/cystic cells and a nonsignificant inhibitory trend in interstitial cells in both males and females (Fig. 1*H* and Supplemental Fig. S2*E*). Interrogation of PKD-associated signaling pathways found that BHB decreased pSTAT3^Y705^ and pERK1/2^T202/Y204^ expression, alongside a reduction in KIM-1 (Supplemental Fig. S1*H*), suggesting that BHB may suppress aberrant activation of these pathways and prevents kidney injury.

We observed a slight increase in animal mass (Supplemental Fig. S1*A*) for all BHB-supplemented Cy/+ groups despite a slight decrease in total calorie consumption (Supplemental Fig. S1*B*). Both 80 and 160 mM BHB groups consumed more water than controls (Supplemental Fig. S1*B*).

The blood glucose of Cy/+ rats was slightly decreased relative to wild-type rats. It was restored to wild-type levels with BHB (Supplemental Fig. S1*E* and Fig. S2*F*), suggesting that the metabolic demand from glycolytic cystic kidneys may lead to systemic blood glucose depletion, similar to previous observations (15, 16, 26). We supplemented BHB as a racemic mixture, so both BHB isomers were measured to report serum BHB levels accurately. Serum BHB levels increased dose dependently following BHB supplementation, with greater concentrations in wild-type rats (Supplemental Fig. S1, *F* and *G*, and Supplemental Fig. S2, *G* and *H*).

### Citrate Administration Ameliorates PKD in a Dose-Dependent Fashion

**Figure 2.**
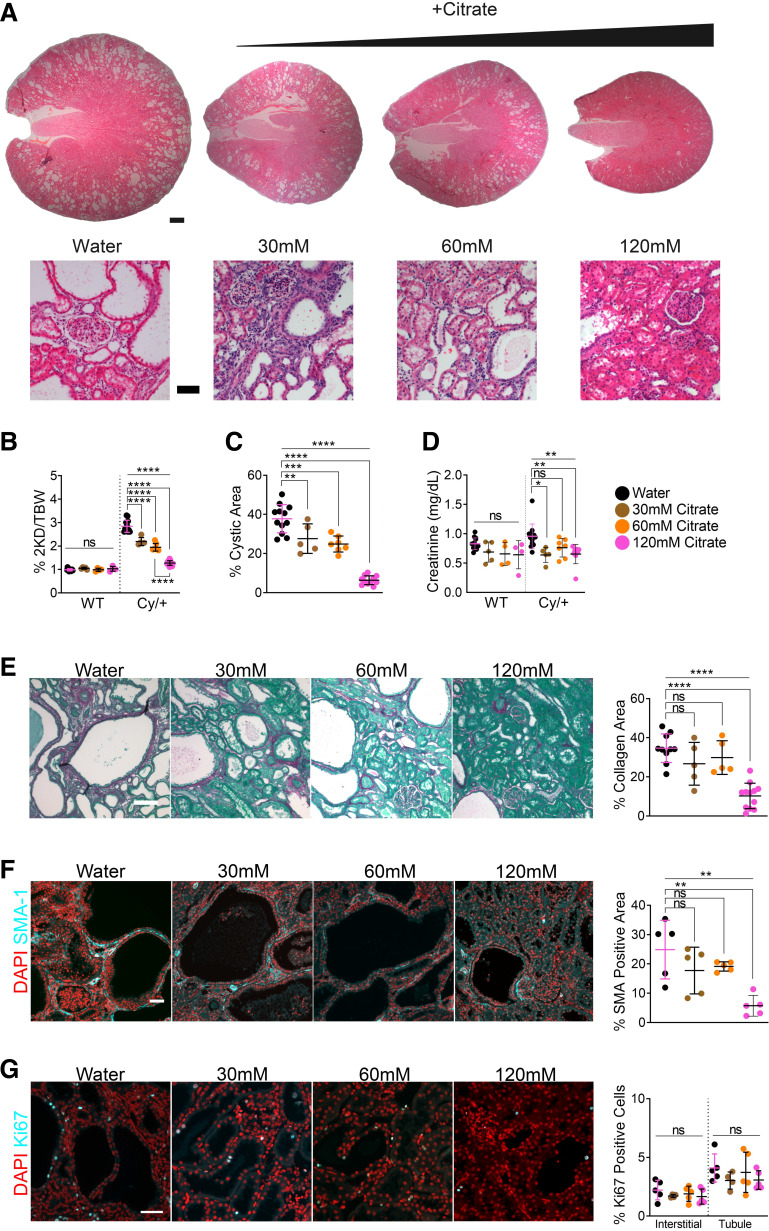
Citrate ameliorates polycystic kidney disease (PKD) in a dose-dependent manner. *A*: hematoxylin and eosin stained kidneys from 8-wk male wild-type and Cy/+ rats supplemented with water or increasing concentrations of citrate in drinking water. Scale bar = 1 mm and 100 µm, respectively. *B*: 2-kidney to the bodyweight of water and citrate-supplemented male wild-type and Cy/+ rats. *C*: cystic area of water and citrate-supplemented male Cy/+ rats. *D*: serum creatinine of male wild-type and Cy/+ rats. *E*: Sirius Red and Fast Green stain and quantification of water and citrate-supplemented male Cy/+ rats. Scale bar = 50 µm. *F*: smooth muscle actin (SMA-1) immunofluorescence and quantification of water and citrate-supplemented male Cy/+ rats. Scale bar = 50 µm. *G*: Ki67 immunofluorescence stain and quantification of water or citrate-supplemented male Cy/+ rats. Scale bar = 50 µm. Wild type: *n* = 12 (Water), *n* = 5 (30 mM citrate), *n* = 4 (60 mM citrate), *n* = 4 (120 mM citrate). Cy/+: *n* = 12 (Water), *n* = 5 (30 mM citrate), *n* = 7 (60 mM citrate), *n* = 12 (120 mM citrate). (Standard deviation and means represented. One-way ANOVA followed by ad hoc Tukey’s test was used for multiple comparisons. *P < 0.05, **P < 0.01, ***P < 0.001, ****P < 0.0001).

To test the dose-dependent response of citrate, we supplemented rats with 120 mM citrate in drinking water and halved the concentration twice to 60 and 30 mM, respectively. Citrate strongly decreased disease progression in a dose-dependent fashion (Fig. 2*A*). All citrate-supplemented rats exhibited decreased two kidney-to-body weight ratios (Fig. 2*B* and Supplemental Fig. S4*A*), cystic indexes (Fig. 2*C* and Supplemental Fig. S4*B*), cyst number (Supplemental Fig. S3*C*), and cyst size (Supplemental Fig. S3*D*), implying that citrate inhibits both cystogenesis and cyst expansion. In addition, citrate improved serum creatinine compared with water alone (Fig. 2*D*).

We measured food and water intake and found that 30 and 60 mM citrate reduced water consumption and that all citrate-supplemented rats consumed fewer calories (Supplemental Fig. S3*B*) without decreasing animal mass (Supplemental Fig. S3*A*).

Blood glucose levels were restored and comparable with the wild-type group with 120 mM citrate (Supplemental Fig. S4*C*) and trended upward with 30 and 60 mM in males (Supplemental Fig. S3*E*). Citrate did not lead to detectable changes in serum d-BHB (Supplemental Fig. S3*F* and Fig. S4*D*).

We investigated collagen deposition (Fig. 2*E* and Supplemental Fig. S4*E*) and myofibroblast expression (Fig. 2*F* and Supplemental Fig. S4*F*) and found that only 120 mM citrate significantly decreased both markers. Ki67 staining showed that citrate did not significantly affect proliferation in males (Fig. 2*G*) but decreased tubule Ki67 positivity in females (Supplemental Fig. S4*G*). Whole kidney analysis found the most potent effects from 120 mM citrate, decreasing pSTAT3^Y705^, and, similar to BHB, reduced KIM-1 expression dose dependently. No impact on pERK1/2^T202/Y204^ expression was observed (Supplemental Fig. S3*G*).

### A Combination of BHB and Citrate Ameliorates PKD in Juvenile Rats

Our previous data using BHB and citrate supplementation suggested that their beneficial effect on disease progression may be caused via distinct mechanisms: *1*) mimicking the state of ketosis and *2*) removal and prevention of injurious tubule microcrystals. We therefore hypothesized that a combination of BHB and citrate (BHB/citrate) may be more efficacious than either administered alone and would allow for reduced dosing.

**Figure 3.**
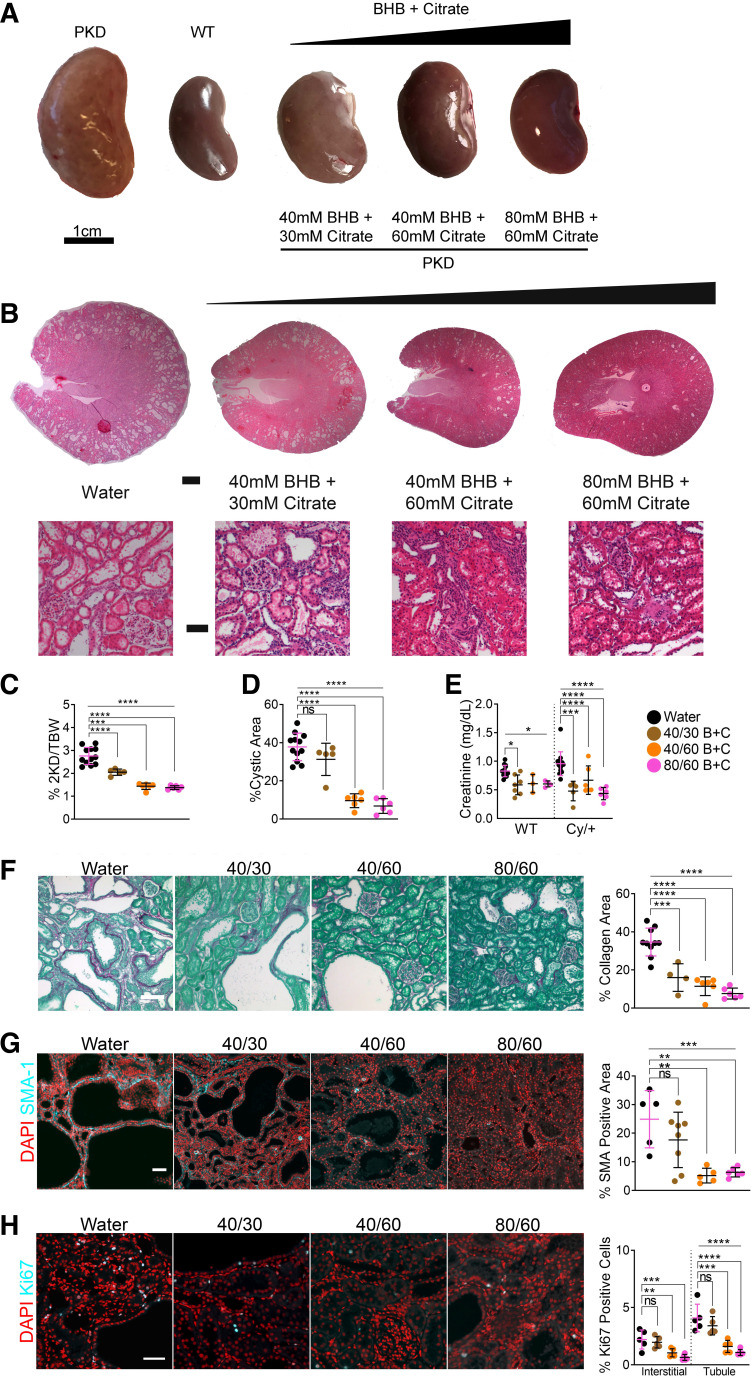
A combination of β-hydroxybutyrate (BHB) and citrate ameliorates polycystic kidney disease (PKD) progression*. A*: gross kidney images of 8-wk water and BHB/citrate-supplemented male wild-type and Cy/+ rats. Scale = 1 cm. *B*: hematoxylin and eosin-stained kidneys from 8-wk male wild-type and Cy/+ rats supplemented with water or increasing concentrations of BHB/citrate in drinking water. Scale bar = 1 mm and 100 µm, respectively. *C*: 2-kidney to the bodyweight of water and BHB/citrate-supplemented male Cy/+ rats. *D*: cystic area of water and BHB/citrate-supplemented male Cy/+ rats. *E*: serum creatinine of water and BHB/citrate-supplemented male wild-type and Cy/+ rats. *F*: Sirius Red and Fast Green stain and quantification of water and BHB/citrate-supplemented male Cy/+ rats. Scale bar = 50 µm. *G*: smooth muscle actin (SMA-1) immunofluorescence and quantification of water and BHB/citrate-supplemented male Cy/+ rats. Scale bar = 50 µm. *H*: Ki67 immunofluorescence stain and quantification of water or BHB/citrate-supplemented male Cy/+ rats. Scale bar = 50 µm. Wild type: *n* = 12 (Water), *n* = 9 (40/30 B+C), *n* = 3 (60/40 B+C), *n* = 4 (80/60 B+C). Cy/+: *n* = 12 (Water), *n* = 5 (40/30 B+C), *n* = 6 (60/40 B+C), *n* = 6 (80/60 B+C). (Standard deviation and means represented. One-way ANOVA followed by ad hoc Tukey’s test was used for multiple comparisons. *P < 0.05, **P < 0.01, ***P < 0.001, ****P < 0.0001).

To test this, we titrated BHB and citrate in combinations determined from the experiments described earlier and administered them in drinking water to rats from *postnatal day 21* to *postnatal day 56* (Fig. 1*A*). These combinations were 40 mM BHB/30 mM citrate, 40 mM BHB/60 mM citrate, and 80 mM BHB/60 mM citrate, labeled as 40/30, 40/60, and 80/60, respectively. BHB/citrate dramatically reduced kidney size (Fig. 3*A*) and the appearance of cysts (Fig. 3*B*). The two kidney-to-body weight ratios (Fig. 3*C*) and cystic area (Fig. 3*D*) were also significantly reduced. Interestingly, both 40/60 and 80/60 combinations dramatically reduced cystic area, similar to BHB or citrate alone (Supplemental Fig. S6, *A* and *E*), suggesting a potential synergistic effect with 40/60 supplementation (Supplemental Fig. S6*F*). We also observed a reduction in cyst number (Supplemental Fig. S5*C* and Fig. S6*E*) and cyst size (Supplemental Fig. S5*D*) in a dose-dependent manner. Like BHB and citrate alone, BHB/citrate reduced serum creatinine levels in Cy/+ rats (Fig. 3*E*) and was more pronounced than with citrate alone (Fig. 2*D*).

Like with BHB alone, blood glucose trended upward with BHB/citrate in Cy/+ rats but without a significant difference in wild-type rats (Supplemental Fig. S5*E*). Again, similar to BHB alone, there was a slight increase in liver mass with BHB/citrate supplementation (Supplemental Fig. S5*A*). Supplementation with 80/60 increased water consumption but did not increase overall caloric intake (Supplemental Fig. S5*B*). BHB/citrate supplementation did not significantly alter steady-state serum total BHB levels (Supplemental Fig. S5, *F* and *G*), suggesting that exogenous BHB is rapidly metabolized.

BHB/citrate supplementation significantly reduced collagen deposition in all BHB/citrate-supplemented concentrations (Fig. 3*F*), was as effective as BHB alone, and was more effective than citrate alone (Supplemental Fig. S6, *B* and *E*). Similarly, myofibroblasts were dramatically reduced with 40/60 and 80/60 (Fig. 3*G* and Supplemental Fig. S6*C*). These effects required less BHB and citrate than when BHB or citrate alone was used, suggesting a potential synergistic activity when combined (Supplemental Fig. S6, *E* and *F*).

We assayed Ki67 and found that 40/60 and 80/60 diminished signals in both interstitial and tubule cells (Fig. 3*H*). BHB or citrate alone showed no significant effect on interstitial cells, with only 160 mM BHB affecting tubule/cystic cells (Supplemental Fig. S6*D*). A comparable impact on total Ki67 (tubule and interstitial) cell positivity was achieved with 40/60 (Supplemental Fig. S6*E*), again implying a synergistic effect (Supplemental Fig. S6*F*). Whole kidney analysis revealed that BHB/citrate decreased pSTAT3^Y705^ in a dose-dependent manner and, unlike BHB or citrate alone, diminished pERK^T202/Y204^ in all BHB/citrate-supplemented groups (Supplemental Fig. S5*H*).

### A Combination of Lower Doses of BHB and Citrate Prevents Kidney Injury and Partially Reverses PKD in Adult Cy/+ Rats

**Figure 4.**
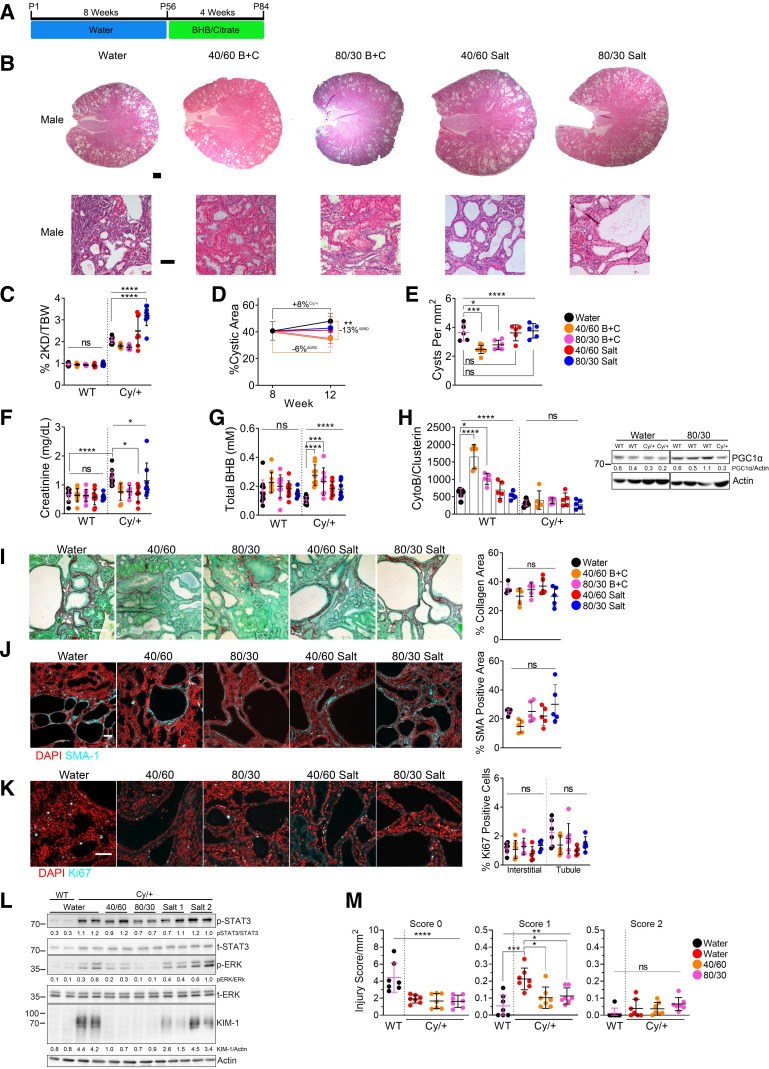
β-Hydroxybutyrate (BHB)/citrate partially reverses polycystic kidney disease (PKD) in adult Cy/+ rats, preventing kidney injury and preserving glomerular health. *A*: timeline of BHB/citrate supplementation in adult rats. *B*: hematoxylin and eosin-stained kidneys from 12-wk male Cy/+ rats supplemented with water, BHB/citrate, or salt in drinking water. Scale bar = 1 mm and 100 µm, respectively. *C*: 2-kidney to the bodyweight of water, BHB/citrate, and salt-supplemented male wild-type and Cy/+ rats. *D*: cystic area changes between 8-wk and 12-wk water, BHB/citrate, and salt-supplemented male Cy/+ rats. *E*: quantification of the number of cysts per mm^2^ from whole kidney sections from water, BHB/citrate, and salt-supplemented male Cy/+ rats. *F*: serum creatinine of water, BHB/citrate, and salt-supplemented male wild-type and Cy/+ rats. *G*: serum total-BHB (l-BHB and d-BHB) values of water, BHB/citrate, and salt-supplemented male wild-type and Cy/+ rats. *H*: mitochondrial number of water, BHB/citrate, and salt-supplemented 12-wk male wild-type and Cy/+ rats, and Western blot of the mitochondrial marker peroxisome proliferator-activated receptor-γ coactivator-1α (PGC1α) in water and 80/30-supplemented male wild-type and Cy/+ rats. All of the samples are on the same blot; the image break is to remove lanes not included in this study. *I*: Sirius Red and Fast Green stain and quantification of water, BHB/citrate, and salt-supplemented male Cy/+ rats. Scale bar = 50 µm. *J*: smooth muscle actin (SMA-1) immunofluorescence and quantification of water, BHB/citrate, and salt-supplemented male Cy/+ rats. Scale bar = 50 µm. *K*: Ki67 immunofluorescence stain and quantification of water, BHB/citrate, and salt-supplemented male Cy/+ rats. Scale bar = 50 µm. *L*: Western blot of whole kidney lysates from water, BHB/citrate, and salt-supplemented male wild-type and Cy/+ rats. (Salt 1 = 40/60 Salt, Salt 2 = 80/30 Salt). *M*: glomerular injury scoring per mm^2^ of kidney area from 12-wk-old water male wild-type and Cy/+ rats and male Cy/+ rats supplemented with 40/60 BHB/citrate or 80/30 BHB/citrate. Glomeruli were scored as follows: 0: no obvious morphological changes; normal, 1: morphological change, e.g., changes in shape and structure, 2: morphological changes as well as decreased filling of glomeruli space, increase in distance between Bowman’s capsule and podocin staining. Wild type: *n* = 12 (Water), *n* = 10 (40/60), *n* = 9 (80/30), *n* = 10 (Salt 1), *n* = 7 (Salt 2). Cy/+: *n* = 12 (Water), *n* = 9 (40/60), *n* = 7 (80/30), *n* = 8 (Salt 1), *n* = 9 (Salt 2). (Standard deviation and means represented. One-way ANOVA followed by ad hoc Tukey’s test was used for multiple comparisons. *P < 0.05, **P < 0.01, ***P < 0.001, ****P < 0.0001).

To test whether reduced doses of BHB and citrate may act together to affect existing cystic disease in adult rats, we supplemented animals from *postnatal day 56* to *postnatal day 84* with either 40 mM BHB and 60 mM citrate (40/60) or 80 mM BHB and 30 mM citrate (80/30), respectively (Fig. 4*A*), and compared this with controls that received water or molar equivalent Na^+^/K^+^ solutions derived from sodium and potassium chloride (40/60 salt and 80/30 salt).

Supplementation with BHB/citrate ameliorated PKD in adult male rats, with reduced cystic disease visible in hematoxylin and eosin-stained kidney sections (Fig. 4*B*). BHB/citrate produced a decrease in kidney mass (Supplemental Fig. S8*A*), a nonsignificant reduction in the two kidney-to-body weight ratio (Fig. 4*C*), a partial reversal in cystic area (Fig. 4*D* and Supplemental Fig. S8*B*) and cyst number (Fig. 4*E*), decreased cyst size (Supplemental Fig. S8*C*), and improved serum creatinine (Fig. 4*F*). Only cyst number was significantly reduced in females with BHB/citrate (Supplemental Fig. S7*D*). We observed no effect on blood glucose (Supplemental Fig. S8*D*), but steady-state blood BHB levels slightly increased (Fig. 4*G* and Supplemental Fig. S7*F* and Fig. S8*F*).

Surprisingly, supplementation with salt caused a reduction in cystic area in female Cy/+ rats (Supplemental Fig. S8*B*). However, the decline in cystic area did not accompany improvements in cyst number (Supplemental Fig. S7*D*) or any other measured outcome. This effect is likely explained by the significantly increased water intake induced by salt supplementation (Supplemental Fig. S8*E*). Increased water intake improves PKD in rodents (27, 28) and has been explored in clinical studies (29, 30).

Mitochondrial damage and mitochondrial loss are known features of PKD progression in the Cy+ rat (19). To explore if BHB/citrate could alter metabolism features, we measured mtDNA number to determine if BHB/citrate might promote mitochondrial biogenesis. We found that BHB/citrate did not affect mtDNA number in Cy/+ kidneys but did lead to an increase in wild-type rat kidneys and was accompanied by an increase in the mitochondrial biogenic marker PGC-1α (Fig. 4*H*). This effect was specific to BHB/citrate since salt alone did not affect mitochondrial number.

Collagen deposition did not differ significantly between BHB/citrate-supplemented cohorts, with only a nonsignificant downward trend in 40/60 males (Fig. 4*I*). Similarly, SMA-1-positive cells decreased with 40/60 supplementation but did not reach significance (Fig. 4*J*). This effect was similar to Ki67 signaling, trending downward with BHB/citrate in tubule/cystic cells without reaching significance (Fig. 4*K*).

Precipitation of microcrystals is a substantive source of kidney injury, leading to activation of the innate immune response, including STAT3 phosphorylation in tubule epithelial cells. Whole kidney lysates revealed that both 40/60 and 80/30 diminished pERK^T202/Y204^ and that only 80/30 decreased pSTAT3^Y705^. Both 40/60 and 80/30 reduced the expression of KIM-1, whereas salt alone did not (Fig. 4*L*).

A previous report found that BHB can preserve and support glomeruli during hyperglycemia (31). To this end, we assessed whether BHB/citrate supplementation might similarly improve glomerular health. We labeled glomeruli in cystic kidneys using the glomerular marker podocin and scored glomeruli for injury (Supplemental Fig. S8*G*). We found more injured glomeruli within cystic kidneys than within wild-type controls and that BHB/citrate decreased glomerular injury compared with water alone (Fig. 4*M*) without affecting the total glomeruli number (Supplemental Fig. S8*H*), implying that part of BHB/citrate’s benefit involves preventing glomerular injury.

Overall, the measured reduction in cyst number, cystic area, glomerular sparing, and absence of KIM-1 and pSTAT3^Y705^ suggests that BHB/citrate can effectively prevent kidney injury, thereby slowing PKD disease progression.

### BHB and Citrate Alter Mineral and Citrate Excretion

Improper mineral handling and reduced citrate excretion are common features of advanced chronic kidney disease (32, 33). The Cy/+ rat exhibits progressive kidney function decline, dysfunctional mineral handling, and concomitant muscle and bone wasting (34). The orthologous Ksp-Cre:*Pkd^fl/fl^* mouse also exhibits reduced serum Mg^2+^, Ca^2+^, Na^+^, and PO_4_^3–^ with associated kidney wasting (35). We have previously reported that decreased urinary citrate was associated with more rapid kidney function decline and larger total kidney volume in individuals with ADPKD (5). A recent report using a more extensive patient set also found that hypocitraturia correlates with worse PKD progression (36). The clinical experience suggests that a standard or elevated urinary citrate level may be protective in ADPKD, presumably due to citrate’s beneficial effect on calcium crystal precipitation and kidney stone risk. To this end, we investigated whether BHB and citrate may alter urine composition to exert their beneficial effects.

**Figure 5.**
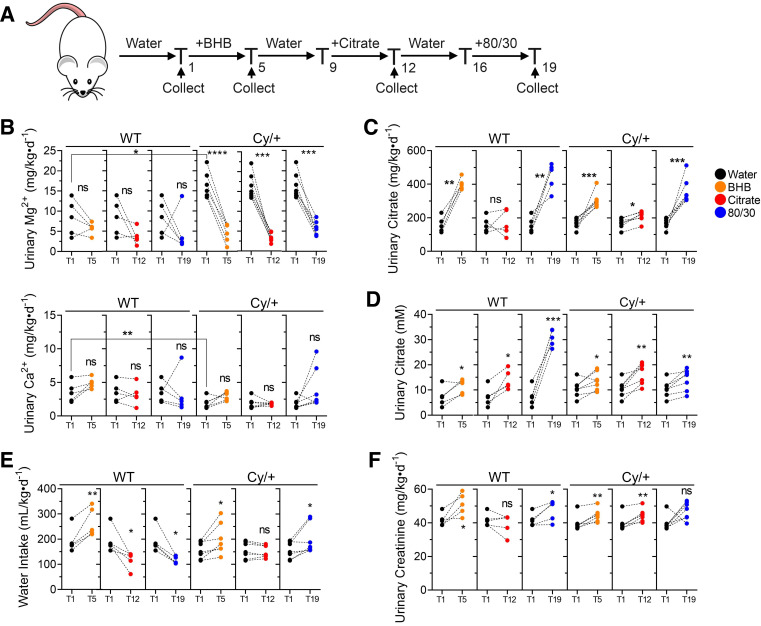
β-Hydroxybutyrate (BHB) and citrate normalize mineral excretion and increase citrate excretion. *A*: treatment scheme for metabolic cage experiments. *B*: change in 24-h urinary magnesium and calcium excretion from BHB, citrate, and BHB/citrate-supplemented male wild-type and Cy/+ rats. *C*: change in 24-h urinary citrate excretion in BHB, citrate, and BHB/citrate-supplemented male wild-type and Cy/+ rats. *D*: change in 24-h urinary citrate concentration in BHB, citrate, and BHB/citrate-supplemented male wild-type and Cy/+ rats. *E*: change in 24-h water intake of BHB, citrate, and BHB/citrate-supplemented male wild-type and Cy/+ rats. *F*: change in 24-h urine creatinine excretion from water, BHB, citrate, and BHB/citrate-supplemented male wild-type and Cy/+ rats. Wild type: *n* = 5; Cy/+: *n* = 7. (Standard deviation and means represented. Paired and unpaired *t* test was used for comparisons. *P < 0.05, **P < 0.01, ***P < 0.001, ****P < 0.0001).

To determine if BHB and citrate alter urine composition, we placed adult Cy/+ rats in metabolic cages for 24-h urine collections following 3 days with 160 mM BHB, 120 mM citrate, or 80 mM BHB/30 mM citrate (80/30) with a 3-day washout period in between each treatment (Fig. 5*A*). Urine was collected and analyzed using UPLC and Inductively Coupled Plasma-Optical Emission Spectrometry, with the results shown in Table 1 detailing mineral and metabolite excretion.

**Table 1.** Metabolic cage experiment animal and urine parameters

	Wild Type				Cy+			
	Water	BHB	Citrate	BHB/Citrate	Water	BHB	Citrate	BHB/Citrate
*n*	5	5	5	5	7	7	7	7
Animal mass, g	278.5 ± 6.0	298.0 ± 6.9	317.0 ± 8.7	345.0 ± 9.9	281.7 ± 10.2	286.7 ± 17.8	275.2 ± 27.8	315.6 ± 23.3
Water intake, mL/kg/day	194.5 ± 49.7	267.8 ± 56.7**	117.3 ± 32.8*	116.0 ± 13.8*	155.5 ± 33.1	201.0 ± 62.1*	151.1 ± 24.0	200.1 ± 59.6*
Food intake, g/kg/day	80.4 ± 4.2	70.3 ± 6.1	59.2 ± 6.8	56.3 ± 11.2	58.7 ± 10.1	67.6 ± 6.3	51.3 ± 10.2	67.1 ± 12.3
BHB intake, µmol/g/day	N/A	42.2 ± 8.9	N/A	9.1 ± 1.1	N/A	33.5 ± 11.3	N/A	15.8 ± 4.7
Citrate intake, µmol/g/day	N/A	N/A	14.0 ± 3.9	3.5 ± 0.4	N/A	N/A	18.0 ± 2.9	6.0 ± 1.8
Urine volume, mL/kg/day	128.7 ± 45.6	191.1 ± 44.8*	61.6 ± 16.9*	75.9 ± 9.3*	87.6 ± 16.5	124.6 ± 44.6*	81.9 ± 18.6	123.5 ± 57.0
Urine creatinine, mg/kg/day	41.8 ± 3.9	51.1 ± 6.5*	39.2 ± 6.0	47.3 ± 6.2*	39.8 ± 4.5	44.5 ± 3.8**	34.3 ± 5.0**	47.3 ± 5.2
Urine creatinine, mg/dL	35.1 ± 10.0	28.1 ± 7.8	71.7 ± 10.8**	58.3 ± 12.6*	46.3 ± 6.7	38.6 ± 10.5	50.8 ± 15.5	39.2 ± 15.5
Potassium consumption, mg/kg/day	885.0 ± 46.2	1374.0 ± 156.1	1406.4 ± 280.9	939.5 ± 153.8	645.3 ± 111.6	1195.1 ± 190.5	1536.8 ± 172.8	1289.7 ± 269.1
Sodium consumption, mg/kg/day	241.4 ± 12.6	811.9 ± 132.4ψ	177.6 ± 20.3	299.2 ± 46.3	176.0 ± 30.4	653.9 ± 151.9ψ	153.9 ± 30.5	425.8 ± 94.1ψ
Potassium excretion, mg/kg/day	320.9 ± 43.1	521.9 ± 104.4**	686.0 ± 264.0	366.0 ± 191.2	281.7 ± 65.8	362.5 ± 74.1	481.3 ± 28.0**	397.8 ± 66.0**
Sodium excretion, mg/kg/day	96.4 ± 12.6	370.0 ± 52.9**	68.9 ± 28.1	129.0 ± 68.5	69.7 ± 24.0[*]	204.3 ± 71.6**	49.8 ± 9.6*	130.3 ± 38.6**
Magnesium excretion, mg/kg/day	8.3 ± 4.4	5.7 ± 1.5	3.6 ± 2.0	4.6 ± 5.1	16.3 ± 3.2[*]	4.6 ± 2.3ψ	3.4 ± 1.1***	5.5 ± 1.8***
Calcium excretion, mg/kg/day	3.6 ± 1.5	4.9 ± 0.8	3.2 ± 1.6	3.3 ± 3.1	1.8 ± 0.8[**]	2.9 ± 0.6	1.8 ± 0.2	4.1 ± 3.1
Potassium excretion, mM	70.4 ± 26.4	74.6 ± 26.7	291.5 ± 103.9	124.5 ± 61.9	86.0 ± 29.3	78.7 ± 29.5	157.0 ± 35.1	91.6 ± 24.1
Sodium excretion, mM	35.7 ± 13.6	88.4 ± 24.6	51.0 ± 20.9	74.2 ± 36.8	36.4 ± 16.1	72.6 ± 32.1	27.0 ± 5.5	48.7 ± 9.2
Magnesium excretion, mM	3.1 ± 1.9	1.3 ± 0.5	2.4 ± 1.1	2.4 ± 2.6	8.0 ± 2.5[*]	1.7 ± 1.0	1.7 ± 0.6	2.2 ± 1.0
Calcium excretion, mM	0.8 ± 0.5	0.7 ± 0.2	1.3 ± 0.5	1.1 ± 1.0	0.5 ± 0.3	0.6 ± 0.1	0.6 ± 0.2	0.8 ± 0.2
Urine citrate, mM	7.3 ± 3.9	11.4 ± 2.8*	14.0 ± 3.8*	30.6 ± 3.3***	10.5 ± 3.3	13.6 ± 3.6*	16.6 ± 16.6**	14.1 ± 4.2**
Urine citrate, mg/kg/day	158.8 ± 46.7	401.0 ± 34.4**	169.8 ± 76.2	447.5 ± 80.0**	169.2 ± 30.3	304.8 ± 48.6***	209.5 ± 31.1*	359.4 ± 75.8***

Analysis of 24-h metabolic cage rat experiments. Rats were supplemented with water, 160 mM β-hydroxybutyrate (BHB), 120 mM citrate, or 80 mM/30 mM BHB/citrate combined. Values represent mean and standard deviation. Paired and unpaired *t* tests are used for analyses when appropriate. *P < 0.05; **P < 0.01; ***P < 0.001; ψ*P* < 0.0001. Parentheticals indicate the statistical difference between wild type and Cy+ rats.

The most striking difference between wild-type and Cy/+ rats was that Cy/+ rats exhibited significant hypermagnesuria and reduced calcium excretion. Elevated urine magnesium excretion was normalized to wild-type levels following administration of BHB, citrate, or 80/30 (Fig. 5*B*). BHB supplementation increases calcium excretion in Cy/+ rats and coincides with a significant increase in citrate excretion and urinary citrate concentration in most individuals (Fig. 5, *C* and *D*). The addition of BHB generally increased water intake in cystic rats, whereas citrate did not (Fig. 5*E*). BHB, citrate, and 80/30 generally increased creatinine excretion in most animals (Fig. 5*F*).

## DISCUSSION

In this study, we built upon our previous research. We more closely investigated BHB, the principal ketone produced during ketosis, and citrate, the eponymous tricarboxylic acid (TCA) cycle intermediate, alone and in combination for their ability to alter PKD progression. We hypothesized that combining BHB and citrate may ameliorate disease progression by simultaneously acting on distinct mechanisms that cause PKD progression, namely, mimicking the effects of ketosis and preventing kidney injury by tubule microcrystals. A limitation of this study was the use of the nonorthologous *Anks6* rat model of kidney disease (Cy/+). This model exhibits proximal tubule-derived cysts and many human chronic kidney disease features. While translationally limiting to ADPKD, these disease features (e.g., the propensity to form microcrystals) make its inclusion in this study relevant.

Our study found that both BHB and citrate very effectively halt the progression of PKD in juvenile rats, and a combination of BHB and citrate recapitulated these findings with decreasing amounts of BHB and citrate. We found that early supplementation in juvenile rats dramatically slows the progression of cystic disease during the time window of most rapid progression in this model. In contrast, adult Cy/+ rats exhibit little additional cystic progression and serve as a model of fully established PKD. Remarkably, treatment with BHB and citrate in adult rats resulted in partial disease reversal, including significant reductions in signs of kidney injury, total cystic area, and total cyst number. This aligns with other observations, including our own, that reversing existing renal cystic disease is possible (20, 37, 38). However, since we did not test BHB or citrate alone in adult rats, we cannot speak to the direct comparative effect of combining BHB and citrate.

Our experiments showed increased creatinine excretion following supplementation with BHB and citrate, suggesting improved renal function. Increased creatinine clearance is consistent with human trials in healthy individuals, wherein intravenous BHB administration increases glomerular filtration independent of an alkali load, putatively via a macula densa feedback mechanism (39). We also found that BHB/citrate prevented glomerular injury in adult rats, similar to a previous report showing improvement of glomerular health with BHB in streptozotocin-induced diabetic nephropathy (31). Our human studies in ADPKD have suggested that ketogenic metabolic therapy increases kidney function (21, 40, 41). Ketogenic metabolic therapy also effectively reduces hypertension (42), arguing against hyperfiltration due to vasoconstriction in our experiments.

Sodium chloride supplementation is known to accelerate the progression of PKD in Cy/+ rats (43). Yet, sodium/potassium BHB salts did not exacerbate disease progression in our study. Instead, they may confer protection from sodium-induced injury, similar to prior reports using butanediol to increase BHB (44). In addition, exogenous BHB was shown to be consumed primarily by the heart and kidney (45), decreasing heart glucose uptake and improving cardiac output (46), and may inhibit any deleterious effects of high salt intake in the context of chronic kidney disease.

We should note that sodium and potassium were delivered as chloride salts to our control animals, and this increase in Cl^–^ may promote fluid secretion into cyst lumens via the activity of the cystic fibrosis transmembrane conductance regulator protein, a known contributor to PKD progression in the Cy/+ rat (47, 48). Both the 40/60 salt and 80/30 salt controls showed an increase in overall kidney size in this way, implicating that Cl^–^ may have exacerbated the disease.

We observed improved serum creatinine in 40/60 salt rats, likely secondary to those animals’ significant increase in water intake. However, the improvement with BHB/citrate supplementation was accompanied by a slight decrease in water intake, arguing against any beneficial effect on creatinine by BHB/citrate being solely due to increased water intake. There were, however, significant differences in feeding behavior between juvenile BHB-, citrate-, and BHB/citrate-supplemented groups (Supplemental Fig. S1*B*, Fig. S3*B*, and Fig. S5*B*). Increasing BHB supplementation correlated with increased water intake, likely caused by the coincident increase in sodium. In support of this hypothesis, the lowest BHB (40 mM)-supplemented animals did not increase water intake and consumed similar amounts of sodium to water-only rats. Interestingly, all BHB-supplemented juveniles consumed comparably reduced calories, implying that BHB may have an anorectic effect even at the lowest dose tested. Citrate alone also produced a similar reduction in calories while reducing water intake similarly between all citrate dosages. This effect of citrate was also observed in our metabolic cage experiments when rats were given citrate alone over 4 days. Citrate-supplemented water is slightly sour to the taste and may cause an aversion until animals are fully habituated. The taste would explain the reduced water intake but not the calorie reduction. This is important since our laboratory and others have demonstrated that caloric reduction beneficially alters PKD progression (49, 50). Although overall calorie intake is decreased with BHB and citrate alone, when combined, this effect vanishes. We interpret these data to mean that the effect of BHB/citrate combined is not simply an artifact of caloric restriction or increased water intake but is caused by an intrinsic property of the combination.

We propose that the beneficial effects of BHB/citrate predominantly come from preventing kidney injury, putatively caused by tubule microcrystals and their subsequent activation of the injury response. This is supported by the measurable reduction in cystogenesis and injury-associated markers (e.g., pSTAT3, KIM-1, SMA-1, collagen, and Ki67) following BHB/citrate supplementation.

BHB is known to exert pleiotropic effects apart from its role as an energy molecule (51), with many of these effects overlapping with those of citrate. Both participate in the TCA cycle, fatty acid synthesis, act as signaling molecules to affect gene regulation, alter energy production, and inhibit inflammation (52, 53). We found that BHB alone, the highest dose of citrate alone, and BHB/citrate strongly inhibited collagen deposition while decreasing SMA-1 and Ki67 expression. It is known that the inflammasome-related protein NOD-, LRR- and pyrin domain-containing protein 3 (NLRP3) is activated in tubule epithelial cells in response to microcrystal-induced kidney injury (54) and is a requirement for collagen deposition (55). NLRP3 is directly inhibited by BHB (53), meaning that BHB/citrate could act upstream and downstream of microcrystal-induced injury, inhibiting microcrystal formation and any subsequent injury response. This makes NLRP3 signaling modification a strong candidate for the mechanism of action of BHB/citrate.

BHB and citrate were delivered as salts, meaning alkali is likely to contribute beneficial effects as PKD kidneys are known to possess an acidic microenvironment. In the Cy/+ rat, this can be exacerbated by ammonium chloride and improved by sodium bicarbonate (56). Bicarbonate supplementation improves outcomes in chronic kidney disease (57), and decreased serum bicarbonate levels are associated with faster progression of PKD (58). Tanner et al. used citrate supplementation and found a dramatic improvement in the Cy/+ rat, concomitantly increasing urine pH. Notably, citrate converts into bicarbonate in the kidney, acts as a glycolysis inhibitor (59), binds excess calcium to prevent kidney stone formation, and putatively cystogenesis (5). Each of these mechanisms may contribute to citrate’s effect but are beyond the scope of the study.

Disrupted mitochondrial energetics and decreased mitochondrial number (19) are features of PKD (26, 60). To our knowledge, we report here that BHB/citrate increases mtDNA number in kidneys for the first time. Whether BHB/citrate also increases mitochondrial health in these kidneys (e.g., via increased aerobic capacity or energy production) remains to be determined. This effect alone could impact metabolic health and warrants further study.

Finally, we examined urine parameters following BHB and citrate supplementation to determine if mineral composition or kidney output was affected. We found that BHB/citrate supplementation reduced markers associated with increased microcrystal formation by normalizing mineral excretion and increasing urinary citrate. Decreased citrate excretion has been associated with increased total kidney volume and reduced estimated glomerular filtration rate (5), whereas increased citrate excretion is associated with slower disease progression (36). It may be that BHB/citrate confers benefits by increasing urinary citrate and lowering the potential of injury from microcrystals, a putative trigger of cystogenesis. However, since we did not fully assess all parameters associated with lithogenic risk, we cannot fully evaluate the effect of BHB/citrate in that respect. Recently, it was reported that tolvaptan decreased markers of lithogenesis in patients with ADPKD after 1 yr of treatment with the drug (61). It is tempting to speculate that much of the benefit of tolvaptan, a strong aquaretic causing dilute urine, may come from preventing kidney injury due to microcrystal formation rather than solely its action on the vasopressin receptor.

Altogether, we have shown that a combination of BHB and citrate reduces PKD progression in both adult and juvenile models of kidney disease by altering cellular signaling to prevent kidney injury, reducing proliferation and fibrosis, improving kidney health by increasing creatinine excretion, preserving glomerular health, and normalizing mineral excretion and increasing urinary citrate. A combination of BHB and citrate is preferred over the use of one or the other alone. First, the combination shows a synergistic effect in juveniles by reducing markers of cystic disease using less BHB and citrate. Second, combining the two compounds reduces the overall salt load, as citrate can partially substitute the effects of BHB and be administered as citric acid.

BHB and citrate are widely available, affordable, and categorized as Generally Recognized as Safe (GRAS) in the United States. Both have a long history of use in food and as supplements, with neither producing substantial negative safety signals. This makes BHB and citrate attractive for the management of individuals with ADPKD. A novel medical food product, KetoCitra, based on BHB and citrate, has already been used in conjunction with a diet and lifestyle intervention program (41), and several clinical trials are forthcoming to investigate the short- and long-term effects in ADPKD.

### Perspectives and Significance

In clinical practice, ADPKD is known to be relentlessly progressive. The sole approved drug for ADPKD only slows this progression. Here, we show that supplementation with a combination of two simple, endogenous compounds, BHB, and citrate, not only dramatically slowed PKD progression in juvenile rats but even partially reversed existing cystic disease in adult rats. This result is highly significant because the observed effect size with BHB/citrate supplementation far exceeds the effect size of pharmacological intervention with vasopressin receptor antagonists in PKD rodent models that underpinned the development of tolvaptan as an approved therapy for ADPKD. Given that BHB and citrate are safe and widely used supplements and food additives, these results suggest that BHB/citrate supplementation may benefit human ADPKD without the side effects and toxicities associated with current pharmacological therapy.

## DATA AVAILABILITY

Data will be made available upon reasonable request.

## SUPPLEMENTAL DATA

10.6084/m9.figshare.c.6927814 Supplemental Figs. S1–S8: https://doi.org/10.6084/m9.figshare.c.6927814.

10.6084/m9.figshare.24556129 Supplemental Animal Use Table: http://doi.org/10.6084/m9.figshare.24556129.

## GRANTS

This work was supported by the National Institutes of Health under Grants R01DK109563 and R01DK124895 and the United States Department of Defense Grant W81XWH2010827 (to T.W.) and gifts from the Amy P. Goldman Foundation and the Jarrett Family Fund to the University of California-Santa Barbara to support the work of T.W. The MRL Shared Experimental Facilities are supported by the MRSEC Program of the National Science Foundation (NSF) under Award DMR 1720256, a member of the NSF-funded Materials Research Facilities Network (www.mrfn.org).

## DISCLOSURES

T.W. and J.A.T. are inventors on issued and pending patents filed by the University of California-Santa Barbara related to PKD; T.W. is a shareholder and president, J.A.T. is a shareholder and employee of Santa Barbara Nutrients, Incorporated; and T.W. was on the scientific advisory board of Chinook Therapeutics, has received research funding from Chinook Therapeutics and speaker fees from Otsuka.

## AUTHOR CONTRIBUTIONS

J.A.T., N.H., and T.W. conceived and designed research; J.A.T., N.H., D.A.A., T.A., B.K., J.R., S.A., and T.W. performed experiments; J.A.T., N.H., D.A.A., T.A., B.K., J.R., and S.A. analyzed data; J.A.T., N.H., and T.W. interpreted results of experiments; J.A.T., N.H., D.A.A., T.A., B.K., and J.R. prepared figures; J.A.T., N.H., and T.W. drafted manuscript; J.A.T., N.H., D.A.A., and T.W. edited and revised manuscript; J.A.T., N.H., and T.W. approved final version of manuscript.
